# Albumin–Bilirubin Grade as a Valuable Predictor of Recurrence and Prognosis in Patients with Hepatocellular Carcinoma Following Radiofrequency Ablation

**DOI:** 10.3390/cancers16244167

**Published:** 2024-12-13

**Authors:** Chang Hun Lee, Ga Ram You, Hoon Gil Jo, Chung Hwan Jun, Eun Young Cho, In Hee Kim, Sung Kyu Choi, Jae Hyun Yoon

**Affiliations:** 1 Department of Internal Medicine, Jeonbuk National University Medical School and Research Institute of Clinical Medicine of Jeonbuk National University Hospital-Jeonbuk National University Medical School, Jeonju 54907, Republic of Korea; chleemd@jbnu.ac.kr (C.H.L.); ihkimmd@jbnu.ac.kr (I.H.K.); 2 Division of Gastroenterology, Department of Internal Medicine, Chonnam National University Hwasun Hospital, Hwasun 58128, Republic of Korea; rugaram27@daum.net; 3 Department of Internal Medicine, Wonkwang University College of Medicine, Iksan 54538, Republic of Korea; jojo420600@gmail.com (H.G.J.); 69-70@hanmail.net (E.Y.C.); 4 Division of Gastroenterology, Department of Internal Medicine, Chonnam National University Medical School, Gwangju 61469, Republic of Korea; estevanj@naver.com (C.H.J.); choisk@chonnam.ac.kr (S.K.C.)

**Keywords:** hepatocellular carcinoma, radiofrequency ablation, albumin–bilirubin grade, recurrence, prognosis

## Abstract

**Background/Objectives:** Radiofrequency ablation (RFA) is an important local treatment for hepatocellular carcinoma (HCC). This study aimed to evaluate the characteristics of tumor recurrence after RFA and analyze predictors of tumor recurrence and survival in patients with HCC. **Methods:** We retrospectively reviewed data from treatment-naïve patients with HCC who underwent RFA for HCC treatment between 2008 and 2017 at four tertiary hospitals in South Korea. **Results:** A total of 636 patients with HCC treated with RFA were enrolled in the study. The mean age was 66.3 ± 10.4 years, with 75.0% of patients being male. Most patients (96.7%) had underlying liver cirrhosis, and viral hepatitis (types B and C) accounted for most cases. The average maximum tumor size was 2.2 ± 0.9 cm, with 84.3% of tumors being single lesions. During the follow-up period, 331 patients experienced recurrence, with 95.5% of cases being intrahepatic and one-fifth occurring at the RFA site. Most patients underwent RFA or transarterial chemoembolization as subsequent therapy for recurrence. Multivariate analysis revealed that age, the albumin–bilirubin (ALBI) grade, and Child–Pugh class B status were independent factors associated with tumor recurrence. Only the ALBI grade was significantly associated with mortality. Additionally, the ALBI grade differentiated between recurrence-free survival and overall survival in the Kaplan–Meier survival curve. **Conclusions:** The ALBI grade was independently associated with tumor recurrence and prognosis in patients with HCC following RFA. This grading system can help clinicians identify high-risk patients, optimize treatment strategies, and enhance patient care.

## Simple Summary

Hepatocellular carcinoma (HCC) remains a significant clinical challenge, with limited progress in reducing recurrence rates and improving patient survival. Selecting appropriate treatment modalities for HCC requires careful consideration of the risks and benefits associated with each option. In this study, we investigated factors influencing recurrence and prognosis in treatment-naïve patients with HCC undergoing radiofrequency ablation (RFA). Our analysis identified age, Child–Turcotte–Pugh class B, and the albumin–bilirubin (ALBI) grade as independent predictors of tumor recurrence. Additionally, the ALBI grade emerged as the sole significant predictor of overall survival. These findings emphasize the importance of incorporating the ALBI grade into the decision-making process to optimize treatment outcomes for patients with HCC undergoing RFA.

## 1. Background

Hepatocellular carcinoma (HCC) is one of the most common and deadly forms of liver cancer worldwide. It ranks as the sixth most common cancer globally and is the third leading cause of cancer-related mortality [1]. HCC predominantly affects middle-aged men, leading to significant economic losses due to its impact on the working-age population [1,2]. Although advancements in the treatment of viral hepatitis B and C have been made, they have not reduced the overall burden of HCC, which is increasingly seen in patients with alcohol-related liver disease and metabolic dysfunction-associated steatotic liver disease [3,4]. Despite various management options and the development of new therapies, the 5-year survival rate remains below 20%, with recurrence rates reaching approximately 70% after curative treatment [5,6,7].

Radiofrequency ablation (RFA) has emerged as a crucial local treatment modality for HCC. It demonstrates comparable efficacy to surgical resection for tumors < 2 cm in size, making it an excellent treatment option for small tumors [8,9,10,11]. Some reports suggest a higher recurrence rate following RFA; however, it is generally acknowledged that there is no significant difference in overall survival between RFA and surgical resection [12,13]. Moreover, RFA offers additional advantages, such as a lower risk of complications, reduced costs, better preservation of normal tissue, and shorter hospital stays [14]. Considering that only 20–25% of patients with HCC are candidates for surgical resection, RFA has become an increasingly valuable treatment option for patients with early-stage tumors [15,16]. As a result, it is strongly recommended in various international guidelines for the treatment of early-stage HCC [14,17,18,19]. The Barcelona Clinic Liver Cancer (BCLC) guidelines recommend RFA for early HCC, particularly in cases of very-early-stage (BCLC stage 0) and early-stage tumors (BCLC stage A) when surgical intervention is not feasible [16].

Despite its effectiveness, tumor recurrence after RFA remains a significant clinical challenge that impacts overall survival and patient prognosis [20,21]. Technical limitations, such as tumors being too deep, located near anatomical boundaries, or having poor sonic visibility, can hinder the success of RFA. Tumors located near major blood vessels, such as the hepatic portal vein or hepatic vein, pose particular difficulties due to the heat sink effect, where blood flow dissipates the heat generated by RFA, reducing its efficacy and potentially leading to higher recurrence rates [22,23]. HCC is known for its high recurrence rate and poor survival outcomes, with a 5-year recurrence rate approaching 70% even after successful initial treatment [24]. Therefore, understanding the factors influencing recurrence and survival is crucial for improving patient management and outcomes.

This study aimed to evaluate the clinical factors associated with tumor recurrence and prognosis in patients undergoing RFA for HCC. By applying recurrence and prognostic factors proposed in previous studies, we aimed to determine which predictors are clinically relevant in a real-world setting. Identifying these predictors will help improve the stratification of at-risk patients, tailor follow-up strategies, and optimize therapeutic interventions to reduce the risk of recurrence and enhance overall survival.

## 2. Materials and Methods

### 2.1. Participants

In this cohort study, we reviewed the medical records of patients with HCC treated between January 2008 and December 2017 at four tertiary hospitals: Chonnam National University Hospital, Gwangju; Hwasun Chonnam National University Hospital, Chonnam; Jeonbuk National University Hospital, Jeonju; and Wongwang University Hospital, Iksan, South Korea. Eligible patients were treatment-naïve, aged > 18 years, and had undergone RFA with complete ablation after being diagnosed with HCC within the Milan criteria. Patients were excluded if they received concurrent treatment with transarterial chemoembolization (TACE) or had a follow-up duration of less than 180 days. The participant selection flowchart is shown in Supplementary Figure S1. A total of 636 patients were enrolled in this study, and demographic, laboratory, and clinical parameters were assessed and analyzed.

### 2.2. HCC Diagnosis and RFA Procedure

HCC was diagnosed based on guidelines by the Korean Liver Cancer Study Group [17,25]. The disease stage was evaluated using both the modified Union for International Cancer Control (mUICC) staging criteria and the BCLC classification system.

Initial tumor size was measured by performing ultrasonography before RFA, and the ablation strategy, power of the generator, and electrode placement were determined by the operator, considering factors such as tumor size, location, and the manufacturer’s recommendations. Ablations were expanded or overlapped to ensure comprehensive tumor coverage, aiming for a safety margin of at least 0.5 cm whenever feasible. Artificial ascites were introduced before RFA to create an insulating layer between the tumor and adjacent vital organs when tumors were located in unfavorable positions. After the lesions were ablated, the ablation path was cauterized to prevent tumor seeding and hemorrhage during the procedure.

A computed tomography (CT) scan was performed immediately after RFA. Complete ablation was defined as the absence of arterial contrast enhancement or portal venous washout within the ablation zone. In cases of incomplete ablation, additional ablation was conducted within 1 or 2 days. Follow-up testing was performed within 1–3 months at the discretion of the researcher.

### 2.3. Follow-Up and Definition of Recurrence

Patients were followed up for clinical assessments and imaging studies. Recurrence was defined as the development of new tumor tissue observed on follow-up imaging after the tumor was previously considered completely ablated. Local tumor progression was defined as the appearance of tumor foci with arterial enhancement and portal or delayed washout at the edge of the ablation zone on contrast-enhanced imaging. Intrahepatic recurrence refers to the regrowth of a previously treated non-target tumor or the emergence of a new tumor within the liver, excluding local progression. Extrahepatic metastasis is defined as the presence of new HCC that appears outside the liver.

### 2.4. Statistical Analysis

Data are expressed as means ± standard deviations and frequencies (percentages) for continuous and categorical variables, respectively. Group comparisons of continuous variables were conducted using the *t*-test, whereas categorical variables were compared using the chi-square or Fisher’s exact test when appropriate. Recurrence and overall mortality were analyzed using univariate and multivariate Cox proportional hazard models. Cumulative recurrence-free and overall survival rates were estimated using the Kaplan–Meier method and compared using the log-rank test. Statistical analyses were performed using IBM SPSS Statistics software (version 23.0.0.0; IBM Corporation, Armonk, NY, USA). All significance tests were two-sided, with *p*-values < 0.05 considered statistically significant.

## 3. Results

### 3.1. Demographic and Baseline Clinical Characteristics of the Study Population

The baseline characteristics of the enrolled patients are shown in Table 1. The mean age of the patients was 66.3 years, with three-quarters of them being male. 

Hepatitis B infection was the most common etiology of chronic liver disease, affecting nearly 60% of the population. Most patients (96.7%) had cirrhotic livers, with 8.2% classified as decompensated (Child–Turcotte–Pugh [CTP] grade B). Regarding HCC staging, the majority of patients were in the early stages of HCC (mUICC stage I or II: 95.6%; BCLC stage 0 or A: 98.1%). The mean tumor size was 2.2 cm, and 84.3% of the tumor lesions were single.

When divided into two groups based on recurrence, the recurrence group included older patients and those with differences in blood test results, including platelet count, aspartate aminotransferase, alanine aminotransferase, total bilirubin, and albumin levels. In the recurrence group, a higher proportion of patients had CTP grade B, and albumin–bilirubin (ALBI) grades II or III were more prevalent than ALBI grade I. The baseline characteristics according to the ALBI grade are described in Supplementary Table S1. There were no statistically significant differences between the two groups regarding BCLC and mUICC stages at the index date. However, the maximum tumor size and the sum of tumor sizes were larger in the recurrence group.

### 3.2. Features Related to RFA Procedures

The clinical characteristics of the RFA procedures are shown in Supplementary Table S2. The tumors were predominantly located in the right lobe, accounting for three-quarters of all cases, and the mean ablation time was 12.8 min. RF needle puncture was performed in a single attempt in 91.5% of patients. RFA-related complications occurred in 78 patients (12.3%), with fever and pleural effusion being the most common, occurring together in seven patients (1.1%). Other complications included hematoma, abscess formation, and cholecystitis. Artificial ascites were created in 34.8% of the procedures to facilitate treatment. Overall, 626 patients (98.4%) underwent complete RFA in a single session, whereas the remaining 10 required repeated ablation to achieve complete ablation. When divided into two groups based on recurrence, the mean ablation time was longer in the recurrence group, and the use of artificial ascites was less frequent.

### 3.3. Clinical Characteristics of Patients with Tumor Recurrence After RFA

The overall and recurrence-free survival rates of the study population are shown in Supplementary Figure S2. Among all patients, 333 (52.3%) experienced HCC recurrence during the follow-up period (Table 2). The alpha-fetoprotein (AFP) level increased from an initial 96.0 IU/mL to 557.7 IU/mL at the time of recurrence. Intrahepatic metastasis accounted for 95.5% of all recurrence cases, with rates of tumor recurrence at the hepatic lobe and RFA site occurring in 141 (42.3%) and 67 (20.1%) patients, respectively. The mUICC stage at recurrence was predominantly stage I or II, accounting for 82% of all cases. More than half of the patients underwent RFA or TACE as rescue therapy. The mean recurrence-free survival was approximately 33 months, and the median time to recurrence was 42.9 months.

### 3.4. Factors Associated with the First Recurrence After RFA

**Figure 1:**
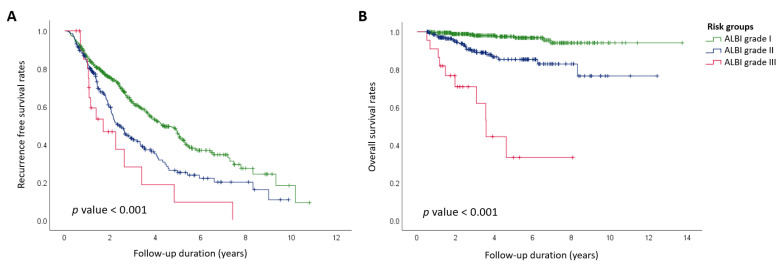
(**A**) Recurrence-free and (**B**) overall survival according to the ALBI grade, as assessed by Kaplan–Meier analysis. ALBI, albumin–bilirubin.

We used a Cox proportional hazards model to analyze independent factors associated with the first recurrence after RFA in patients with HCC, and the results are shown in Table 3. In the univariate analysis, factors associated with recurrence included age, maximum tumor size > 2 cm, non-single tumors, BCLC stage, mUICC stage, ALBI grade, and CTP grade B. In the multivariate analysis, age, ALBI grade, and CTP grade B emerged as independent factors associated with the first recurrence after RFA. The Kaplan–Meier survival analysis, using the log-rank test for recurrence-free survival, indicated that ALBI grades showed statistically significant differences in recurrence after RFA (Figure 1A). We performed a Kaplan–Meier survival analysis based on the ALBI grade according to the type of recurrence. While there was no significant difference in local progression-free survival across ALBI grades, significant differences were observed in intrahepatic and extrahepatic recurrence-free survival (Supplementary Figure S3).

### 3.5. Factors Associated with Mortality After RFA

During the follow-up period, 50 patients (7.8%) died. We analyzed independent factors associated with mortality after RFA in patients with HCC using the Cox proportional hazards model (Table 4). In the univariate analysis, factors such as maximum tumor size > 2 cm, BCLC stage, ALBI grade, and CTP grade B were associated with poor outcomes. In the multivariate analysis, the ALBI grade emerged as the only independent factor associated with mortality after RFA in patients with HCC. The ALBI grade demonstrated a statistically significant difference in mortality after RFA, as shown by Kaplan–Meier survival analysis using the log-rank test (Figure 1B).

## 4. Discussion

HCC remains a significant clinical challenge, with limited progress in reducing recurrence rates and improving patient survival. Selecting appropriate treatment modalities for HCC requires careful consideration of the risks and benefits associated with each option. In this study, we investigated factors influencing recurrence and prognosis in treatment-naïve patients with HCC undergoing RFA. Our analysis identified age, CTP class B, and the ALBI grade as independent predictors of tumor recurrence. Furthermore, the ALBI grade emerged as the sole significant predictor of overall survival, highlighting its critical role in patient prognosis. These findings emphasize the importance of incorporating the ALBI grade into the decision-making process to optimize treatment outcomes for patients with HCC undergoing RFA.

Over the past three decades, RFA procedures have advanced significantly, particularly due to the development of imaging technologies that enable precise targeting and real-time monitoring of ablation procedures [22]. In addition to these imaging improvements, the introduction of novel ablation devices has enhanced efficacy. A key advancement is the no-touch multi-bipolar RFA technique, which mitigates the risk of tumor seeding by using multiple electrodes positioned around the tumor, offering better control over the ablation zone [26]. Moreover, innovations such as artificial ascites and pleural effusion have improved the safety and effectiveness of ablation in challenging anatomical locations and reduced complications related to adjacent structures. Combining RFA with other treatment modalities for HCC offers synergistic effects that enhance both local tumor control and systemic therapeutic outcomes. Despite these technological and procedural advancements, tumor recurrence remains a significant challenge, necessitating continuous research to optimize RFA techniques for improved long-term survival outcomes and reduced recurrence rates.

Many studies have emphasized that advanced tumor stage and poor liver function are significant predictors of tumor recurrence and survival in patients with HCC treated with RFA [27,28]. Recurrence patterns have been analyzed based on local versus distant recurrence and duration to distinguish between early and late recurrences. Tumor size consistently emerges as a critical factor in predicting recurrence; tumors larger than 3 cm, particularly those located near intrahepatic blood vessels or in subcapsular regions, have a higher risk of incomplete ablation and subsequent recurrence [29,30,31]. The number of tumors is also a significant factor, associated not only with early recurrence but also with late recurrence [32,33,34,35,36]. Additionally, periportal HCC carries a higher risk of recurrence due to its proximity to major vascular structures, complicating complete ablation [37]. Impaired liver function, as measured using the Child–Pugh score, is crucial for recurrence outcomes. Patients with compromised liver function face an increased risk of recurrence due to impaired hepatic recovery following RFA and are more susceptible to hepatic decompensation, which can significantly reduce overall survival. Several studies have highlighted the impact of cirrhosis, the Child–Pugh class, albumin levels, and prolonged prothrombin time on recurrence rates [29,32,34,36]. In addition, factors such as age, male sex, high levels of AFP and gamma-glutamyl transferase, and an elevated neutrophil-to-lymphocyte ratio (NLR) have been identified as predictors of recurrence following RFA for HCC [33,38,39].

Several factors identified in previous studies were also associated with HCC recurrence in our study. Increasing age was significantly correlated with a higher risk of recurrence, aligning with the established understanding that older age is a predictor of poor outcomes in many malignancies. Furthermore, liver function, assessed using the Child–Pugh score, plays a critical role in determining both tumor recurrence and patient survival. Our findings revealed that patients with Child–Pugh class B disease had significantly higher recurrence rates than those with Child–Pugh class A, emphasizing the detrimental impact of impaired liver function on post-RFA outcomes. In analyzing other previously reported factors related to recurrence and prognosis—such as tumor size, tumor number, AFP levels, and the NLR—none demonstrated statistical significance in the multivariate analysis. The lack of statistical significance for these risk factors may be due to the limited number of patients with high-risk characteristics in our cohort, potentially reducing statistical power. This highlights the importance of recognizing these risk factors when selecting treatment modalities, as certain cases may have been underrepresented in our analysis, affecting our ability to detect meaningful differences.

In our study, the ALBI grade was the only significant factor related to both recurrence and prognosis. The ALBI score is calculated using the following formula: ALBI = (log_10_ bilirubin [μmol/L] × 0.66) + (albumin [g/L] × −0.085). Based on this score, patients are classified into three grades: ALBI grade I (≤ −2.60), ALBI grade II (> −2.60 to ≤ −1.), and ALBI grade III (> −1.39) [40]. This novel scoring system evaluates liver function based solely on serum albumin and bilirubin levels, demonstrating prognostic value across various liver diseases, irrespective of etiology [41,42]. Compared to the CTP score, the ALBI grade has the advantage of simplicity, as it excludes subjective factors and relies exclusively on two objective indicators. This simplicity makes it a straightforward and reproducible tool for assessing liver function [43]. In the context of HCC, the ALBI grade is a valuable predictor of prognosis and has shown promise across various treatment modalities, including RFA, thereby optimizing treatment outcomes [44,45,46,47,48,49]. Additionally, the ALBI grade has been introduced as a prognostic indicator in other conditions, including chronic heart failure and brain tumors [42].

As various advanced treatment modalities for HCC continue to evolve, a detailed assessment of liver function has become increasingly important. The ALBI grade serves as a reliable indicator of liver function in patients with HCC and aids in prognostic prediction [50,51]. Numerous studies have validated the ALBI grade as a significant factor in predicting recurrence and prognosis across a range of HCC treatment modalities, including surgery, RFA, TACE, systemic chemotherapy, and combination therapies [44,48,49,52,53,54,55]. Furthermore, the ALBI grade plays a crucial role in guiding treatment decisions, with its impact evident even within established prognostic frameworks such as the BCLC and CTP scores [56]. Modified equations, such as modified ALBI, ALBI–triglyceride, and easy ALBI, have also been developed [57], and combinations with other factors, such as the age–male–ALBI–platelet score, the aMAP score, have been used to predict recurrence and prognosis more effectively [52,58,59].

In this study, we confirmed that the ALBI grade is an independent predictor of recurrence and survival in patients with HCC undergoing RFA. Notably, the ALBI grade emerged as the only significant predictor of overall survival, aligning with the results of previous studies and emphasizing its importance. The ability of the ALBI grade to more accurately reflect liver function compared to traditional assessment tools makes it a critical factor in developing treatment strategies for patients with HCC. When analyzed by ALBI grade, we observed significant differences in overall recurrence-free survival. While local tumor progression rates showed no significant differences, intrahepatic recurrence was more frequent in ALBI grades II and III, and extrahepatic metastasis was predominantly observed in ALBI grade III. These findings highlight the need for enhanced monitoring strategies, including careful surveillance for intrahepatic recurrence beyond the RFA-treated site in patients with ALBI grade II or III. Short-term imaging follow-up may be particularly beneficial for these patients. For ALBI grade III patients, who are prone to frequent recurrence and have poor prognoses, alternative modalities, such as liver transplantation, could be considered in selected cases. These findings suggest that incorporating the ALBI grade into clinical practice can enhance the risk stratification of patients with HCC, facilitating personalized treatment plans and improving overall outcomes.

This study demonstrates the utility of the ALBI grade in predicting recurrence and prognosis in HCC patients undergoing RFA, emphasizing its practical application and predictive power in real-world settings. While the retrospective design imposes certain limitations, the study benefits from the inclusion of data from four tertiary hospitals in South Korea, which ensures a diverse patient population and enhances the generalizability of the findings. Additionally, the long-term follow-up period enabled a thorough assessment of the ALBI grade’s prognostic value. By stratifying patients based on the ALBI grade, this study offers a detailed analysis of the associations between recurrence patterns and ALBI grades, providing valuable insights into their clinical implications.

This study has several limitations, primarily due to its retrospective design. Retrospective studies are inherently susceptible to selection bias and have limited control over confounding variables. The data were collected from medical records, which may contain inconsistencies or incomplete information, potentially affecting the accuracy of the results. Furthermore, due to the diagnostic characteristics of HCC, it was not possible to analyze recurrence and prognosis based on the histologic subtype or histological grade, nor their association with the ALBI grade. The small number of patients with ALBI grade III in our study may limit the precision of our findings. Future studies with larger cohorts are needed to validate the prognostic value of the ALBI grade. Additionally, since the study was conducted across multiple tertiary hospitals in South Korea, variations in RFA techniques and the skill levels of practitioners could have influenced the outcomes. Consequently, these findings may not be generalizable to other populations or healthcare settings. To address these limitations, future research should consider prospective study designs that allow for more rigorous control of confounding variables and consistent data collection. Multicenter studies involving diverse populations from different geographical regions could help validate our findings and enhance their generalizability. Furthermore, randomized controlled trials should be conducted to establish stronger causal relationships between the identified predictors and patient outcomes. By addressing these limitations, future research could provide more robust and comprehensive insights into the factors influencing recurrence and prognosis in patients with HCC undergoing RFA.

## 5. Conclusions

This study demonstrates that the ALBI grade is independently associated with tumor recurrence and prognosis in patients with HCC following RFA. These findings underscore the importance of incorporating the ALBI grade into clinical practice for improved prognostic assessment and tailored management of patients post-RFA. By utilizing the ALBI grade, clinicians can more accurately identify patients at higher risk of recurrence and poor outcomes, thereby optimizing treatment strategies and improving patient care.

## Figures and Tables

**Table 1:** Baseline characteristics of enrolled patients.

Characteristics	No Recurrence (n = 303)	Recurrence (n = 333)	Total (n = 636)	*p*-Value
Age	65.4 ± 10.6	67.2 ± 10.2	66.3 ± 10.4	0.032
Male sex	227 (74.9%)	250 (75.1%)	477 (75.0%)	1.000
Etiology of chronic liver disease				
Alcohol	91 (30.0%)	95 (28.5%)	186 (29.2%)	0.742
HBV	178 (58.7%)	198 (59.5%)	376 (59.1%)	0.909
HCV	55 (18.2%)	57 (17.1%)	112 (17.6%)	0.812
NASH	0 (0.0%)	2 (0.6%)	2 (0.3%)	0.521
Cryptogenic	1 (0.3%)	4 (1.2%)	5 (0.8%)	0.428
Presence of liver cirrhosis	291 (96.0%)	324 (97.3%)	615 (96.7%)	0.506
Laboratory results				
WBCs (/mm^3^)	5748.7 ± 2311.8	5775.2 ± 2581.7	5762.6 ± 2454.8	0.892
Hemoglobin (mg/dL)	12.8 ± 1.9	13.1 ± 1.6	13.0 ± 1.7	0.379
Platelet (/mm^3^)	137.6 ± 62.9	120.5 ± 55.7	128.7 ± 59.8	<0.001
Prothrombin time (PT-INR)	1.1 ± 0.2	1.2 ± 0.2	1.1 ± 0.2	0.086
AST (IU/L)	54.8 ± 61.4	68.6 ± 79.8	62.0 ± 71.9	0.015
ALT (IU/L)	36.9 ± 41.2	47.3 ± 69.6	42.4 ± 58.0	0.021
Total bilirubin (mg/dL)	1.0 ± 0.8	1.2 ± 1.6	1.1 ± 1.3	0.017
Albumin (g/dL)	4.1 ± 0.6	3.9 ± 0.6	4.0 ± 0.6	<0.001
Serum AFP (IU/mL)	5.5 [3.0;29.6]	9.8 [4.8;51.2]	7.8 [3.5;38.3]	<0.001
PIVKA-II (mAU/mL)	22.0 [17.0;31.0]	24.0 [17.0;46.0]	23.0 [17.0;36.5]	0.090
CTP grade				0.007
A	288 (95.0%)	296 (88.9%)	584 (91.8%)	
B	15 (5.0%)	37 (11.1%)	52 (8.2%)	
Neutrophil-lymphocyte ratio	4.1 ± 7.3	4.1 ± 5.7	4.1 ± 6.5	0.899
ALBI grade				0.010
I	203 (67.7%)	183 (56.0%)	386 (61.6%)	
II	88 (29.3%)	128 (39.1%)	216 (34.4%)	
III	9 (3.0%)	16 (4.9%)	25 (4.0%)	
BCLC stage				0.093
0	155 (51.2%)	148 (44.4%)	303 (47.6%)	
A	145 (47.9%)	176 (52.9%)	321 (50.5%)	
B	3 (1.0%)	9 (27%)	12 (1.9%)	
mUICC stage				0.229
I	175 (57.8%)	170 (51.1%)	345 (54.2%)	
II	115 (38.0%)	148 (44.4%)	263 (41.4%)	
III	13 (4.3%)	15 (4.5%)	28 (4.4%)	
Maximum tumor size (cm)	1.9 ± 0.6	2.0 ± 0.7	1.9 ± 0.6	0.015
Sum of tumor size (cm)	2.1 ± 0.8	2.3 ± 1.0	2.2 ± 0.9	0.002
Tumor number				0.155
1	264 (87.1%)	272 (81.7%)	536 (84.3%)	
2	35 (11.6%)	53 (15.9%)	88 (13.8%)	
3	4 (1.3%)	8 (2.4%)	12 (1.9%)	
Encapsulated tumor	63 (20.8%)	67 (20.1%)	130 (20.4%)	0.911
Subcapsular tumor	115 (38.0%)	123 (36.9%)	238 (37.4%)	0.855
Follow-up duration (days)	1183.6 ± 805.1	1669.3 ± 947.3	1437.9 ± 914.5	<0.001

Data are expressed as number (percentage) or mean ± standard deviation. AFP and PIKV-II levels are presented as median [25–75% interquartile range]. HBV, hepatitis B virus; HCV, hepatitis C virus; NASH, non-alcoholic steatohepatitis; WBCs, white blood cells; INR, international normalized ratio; AST, aspartate aminotransferase; ALT, alanine aminotransferase; AFP, alpha-fetoprotein; PIVKA-II, protein induced by vitamin K absence or antagonist-II; CTP grade, Child–Turcotte–Pugh grade; ALBI, albumin–bilirubin; BCLC, Barcelona Clinic Liver Cancer; mUICC, modified Union for International Cancer Control.

**Table 2:** Characteristics of patients with first recurrence following RFA.

Characteristics	(n = 333)
AFP level	
Initial	9.8 [4.8;51.2]
Recurrence	6.7 [3.2;44.0]
CTP grade	
A	296 (88.9%)
B	37 (11.1%)
First recurrence site	
RFA site	67 (20.1%)
Same hepatic lobe	141 (42.3%)
Different hepatic lobe	83 (24.9%)
Both hepatic lobe	27 (8.1%)
Extrahepatic area	15 (4.5%)
mUICC stage at recurrence	
Ⅰ	169 (50.8%)
Ⅱ	106 (31.9%)
Ⅲ	35 (10.5%)
Ⅳa	8 (2.4%)
Ⅳb	15 (4.5%)
Rescue treatment modality	
TACE	142 (42.6%)
RFA	126 (37.8%)
TACE + RFA	11 (3.3%)
Surgical resection	11 (3.3%)
Radiotherapy	3 (0.9%)
Liver transplantation	1 (0.3%)
Systemic chemotherapy	2 (0.6%)
Best supportive care	17 (5.1%)
Follow-up loss	20 (6.0%)
Recurrence-free survival, months	33.1 ± 25.3
Median time to recurrence, months	42.9 [18.5–90.2]

Data are expressed as number (percentage) or mean ± standard deviation. AFP levels at initial diagnosis and recurrence, as well as the median time to recurrence, are presented as medians with [25–75% interquartile range]. AFP, alpha-fetoprotein; CTP grade; Child–Turcotte–Pugh grade; RFA, radiofrequency ablation; mUICC, modified Union for International Cancer Control; TACE, transarterial chemoembolization.

**Table 3:** Factors associated with recurrence after RFA using Cox proportional hazards model.

	Univariate Analysis				Multivariate Analysis			
	*p*-Value	HR	Lower CI	Upper CI	*p*-Value	HR	Lower CI	Upper CI
**Age**	**0.022**	**1.013**	**1.002**	**1.024**	**0.045**	**1.012**	**1.000**	**1.024**
Male sex	0.479	1.094	0.853	1.403				
Tumor size, Maximum diameter > 2 cm	0.010	1.334	1.072	1.661	0.074	1.307	0.975	1.751
Non-single tumor (vs. single lesion)	0.034	1.351	1.023	1.785	0.073	1.347	0.973	1.865
BCLC stage (0 vs. A, B)	0.005	0.361	1.096	1.690	0.397	1.233	0.759	2.001
mUICC stage (I vs. II, III)	0.017	1.301	1.049	1.613	0.554	0.871	0.553	1.374
Encapsulated HCC	0.671	0.943	0.722	1.234				
Subcapsular lesion	0.862	0.980	0.785	1.225				
Post-RFA complications	0.644	1.081	0.777	1.505				
Presence of liver cirrhosis	0.702	1.138	0.587	2.208				
Alcoholic cirrhosis	0.400	1.108	0.872	1.407				
**ALBI grade**	**<0.0001**	**1.615**	**1.343**	**1.942**	**0.018**	**1.327**	**1.049**	**1.679**
NLR ≥ 1.55	0.056	1.257	0.994	1.589				
**CTP grade B (vs. A)**	**<0.0001**	**2.378**	**1.684**	**3.356**	**0.010**	**1.901**	**1.173**	**3.082**
AFP ≥ 200 IU/mL	0.270	1.233	0.850	1.791				

BCLC, Barcelona Clinic Liver Cancer; mUICC, modified Union for International Cancer Control; HCC, hepatocellular carcinoma; RFA, radiofrequency ablation; ALBI, albumin–bilirubin; NLR, neutrophil–lymphocyte ratio; CTP grade, Child–Turcotte–Pugh grade; AFP, alpha-fetoprotein; HR, hazard ratio; CI, confidence interval.

**Table 4:** Factors associated with overall survival after RFA using Cox proportional hazards model.

	Univariate Analysis				Multivariate Analysis			
	*p*-Value	HR	Lower CI	Upper CI	*p*-Value	HR	Lower CI	Upper CI
Age	0.704	0.887	0.479	1.645				
Male sex	0.750	1.004	0.977	1.033				
Tumor size, Maximum diameter >2 cm	0.039	1.873	1.031	3.403	0.270	1.597	0.696	3.665
Non-single tumor (vs. single lesion)	0.196	1.556	0.796	3.038				
BCLC stage (0 vs. A, B)	0.034	1.885	1.050	2.386	0.464	1.370	0.590	3.179
mUICC stage (I vs. II, III)	0.117	1.564	0.894	2.733				
Encapsulated HCC	0.265	0.635	0.285	1.411				
Subcapsular lesion	0.655	1.138	0.646	2.004				
Post-RFA complications	0.770	0.871	0.346	2.195				
Presence of liver cirrhosis	0.386	21.176	0.021	21,120.0				
Alcoholic cirrhosis	0.652	1.149	0.627	2.106				
**ALBI grade**	**<0.0001**	**4.796**	**3.179**	**7.235**	**<0.0001**	**3.710**	**2.098**	**6.560**
NLR ≥ 1.55	0.119	1.684	0.875	3.241				
CTP grade B (vs. A)	<0.0001	7.237	3.981	13.154	0.153	1.916	0.785	4.675
AFP ≥ 200 IU/mL	0.131	0.217	0.030	1.576				

BCLC, Barcelona Clinic Liver Cancer; mUICC, modified Union for International Cancer Control; HCC, hepatocellular carcinoma; RFA, radiofrequency ablation; ALBI, albumin–bilirubin; NLR, neutrophil–lymphocyte ratio; CTP grade, Child–Turcotte–Pugh grade; AFP, alpha-fetoprotein; HR, hazard ratio; CI, confidence interval.

## Data Availability

Non-identifiable data will be made available upon reasonable request.

## Supplementary Materials

The following supporting information can be downloaded at: https://www.mdpi.com/article/10.3390/cancers16244167/s1, Figure S1: Participant Selection Flow Chart; Figure S2: (A) Overall survival rates and (B) recurrence-free survival rates among the overall population.; Figure S3: (A) Local progression-free survival rates, (B) intrahepatic recurrence-free survival rates, and (C) extrahepatic recurrence-free survival rates according to the ALBI grade.; Table S1: Baseline characteristics of enrolled patients according to the ALBI grade.; Table S2: Clinical information related to the RFA procedure.

## Abbreviations

RFA	Radiofrequency ablation
HCC	hepatocellular carcinoma
ALBI	albumin–bilirubin
BCLC	Barcelona Clinic Liver Cancer
TACE	transarterial chemoembolization
AFP	alpha-fetoprotein
mUICC	modified Union for International Cancer Control
CT	computed tomography
CTP	Child–Turcotte–Pugh
NLR	neutrophil-to-lymphocyte ratio
