# Supplementary material for: Albumin–Bilirubin Grade as a Valuable Predictor of Recurrence and Prognosis in Patients with Hepatocellular Carcinoma Following Radiofrequency Ablation
Source: Cancers (Basel). 2024 Dec 13;16(24):4167. doi: 10.3390/cancers16244167 (PMC11674869; doi:10.3390/cancers16244167)
Supplement: Supplementary file 1 [file cancers-16-04167-s001.zip › cancers-3298786-supplementary.pdf]

Supplementary Material

Albumin-Bilirubin Grade as a Valuable Predictor of Recurrence and Prognosis in Patients with Hepatocellular Carcinoma Following Radiofrequency Ablation

Chang Hun Lee[1], Ga Ram You[2], Hoon Gil Jo[3], Chung Hwan Jun[4], Eun Young Cho[3], In Hee Kim[1], Sung Kyu Choi[4], Jae Hyun Yoon[4*]

[1]Department of Internal Medicine, Jeonbuk National University Medical School and Research Institute of Clinical Medicine of Jeonbuk National University Hospital-Jeonbuk National University Medical School, Jeonju, Korea

[2]Division of Gastroenterology, Department of Internal Medicine, Chonnam National University Hwasun Hospital, Hwasun, Korea

[3]Department of Internal Medicine, Wonkwang University College of Medicine, Iksan, Korea

[4]Division of Gastroenterology, Department of Internal Medicine, Chonnam National University Medical School, Gwangju, Korea

***Correspondence:**
Jae Hyun Yoon, MD., PhD, Department of Gastroenterology and Hepatology, Chonnam National University Hospital and Medical School, 40 Jebong-ro, 61469, South Korea
zenmake14@gmail.com

Contents

1 Supplementary Figures

2 Supplementary Tables

Supplementary Figure S1. Participant Selection Flow Chart.

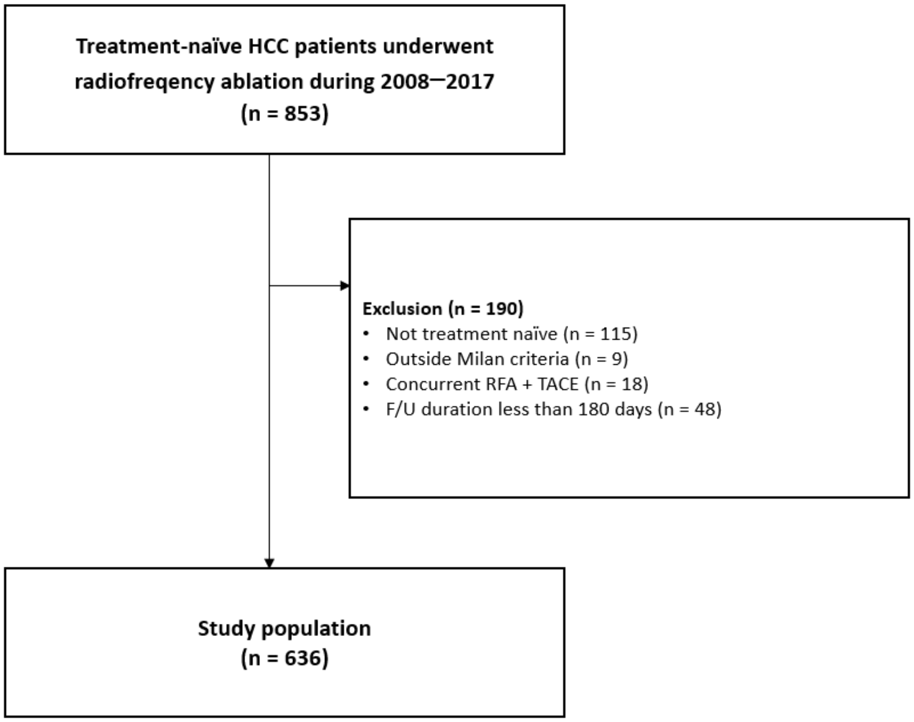

Supplementary Figure S2. (A) Overall survival rates and (B) recurrence-free survival rates among the overall population.

A

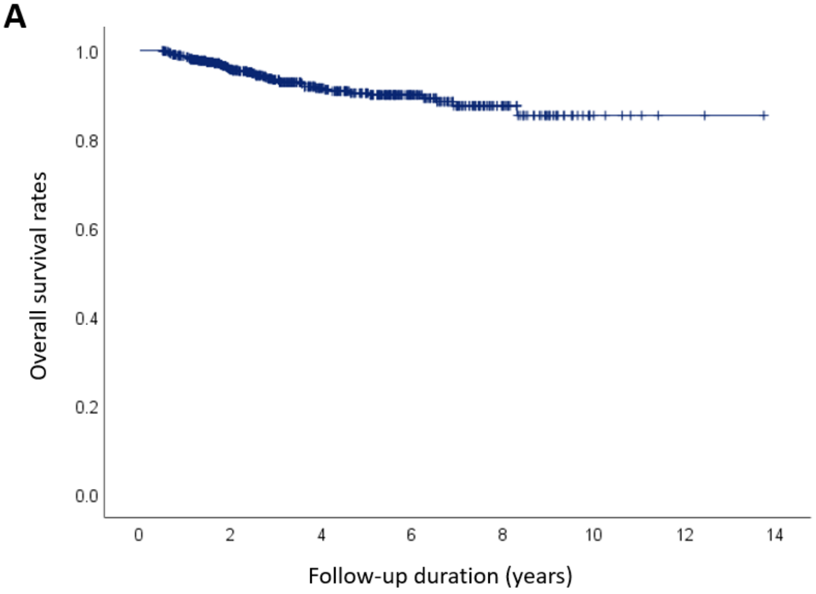

B

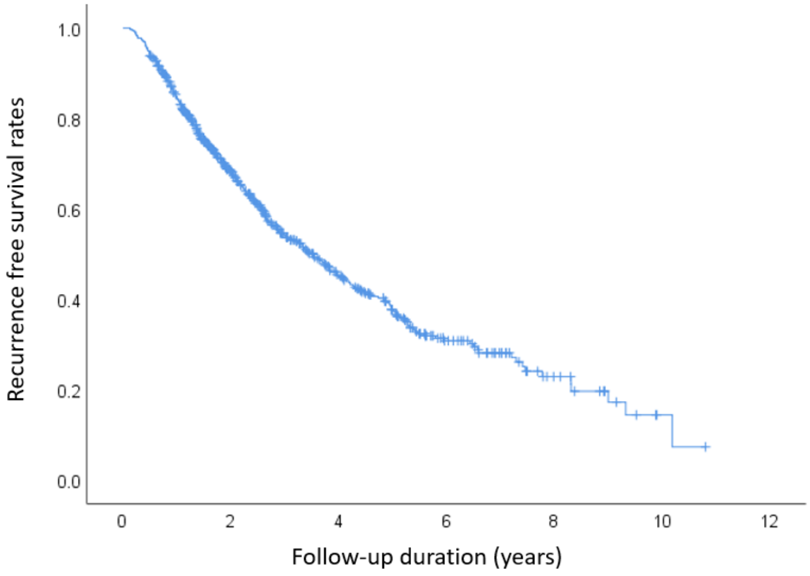

Supplementary Figure S3. (A) Local progression-free survival rates, (B) intrahepatic recurrence-free survival rates, and (C) extrahepatic recurrence-free survival rates according to the ALBI grade. ALBI, albumin-bilirubin.

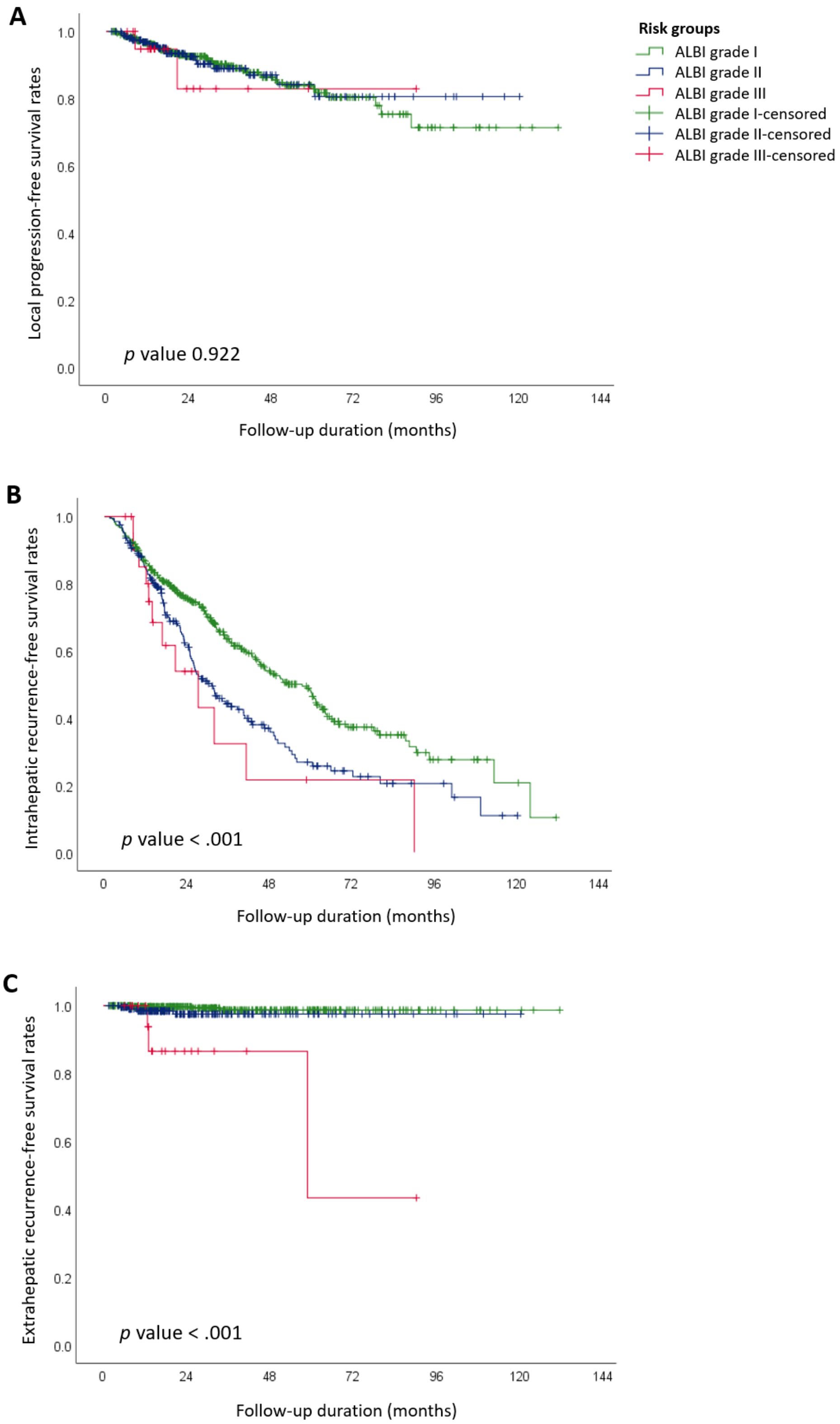

Supplementary Table S1. Baseline characteristics of enrolled patients according to the ALBI grade.

Characteristics	ALBI grade I (n = 386)	ALBI grade II (n = 216)	ALBI grade III (n = 25)	Total (n = 627)	P value
Age	65.9 ± 10.2	67.5 ± 10.9	63.1 ± 9.4	66.3 ± 10.4	0.062
Male sex	307 (79.5%)	143 (66.2%)	20 (80.0%)	470 (75.0%)	0.001
Etiology of chronic liver disease					
Alcohol	91 (23.6%)	82 (38.0%)	13 (52.0%)	186 (29.7%)	< 0.001
HBV	258 (66.8%)	98 (45.4%)	13 (52.0%)	369 (58.9%)	< 0.001
HCV	57 (14.8%)	49 (22.7%)	5 (20.0%)	111 (17.7%)	0.048
NASH	0 (0.0%)	1 (0.5%)	1 (4.0%)	2 (0.3%)	0.002
Cryptogenic	0 (0.0%)	4 (1.9%)	0 (0.0%)	4 (0.6%)	0.022
Presence of liver cirrhosis	366 (94.8%)	215 (99.5%)	25 (100.0%)	606 (96.7%)	0.005
Laboratory results					
WBC (/mm^3)	6086.9 ± 2158.9	5218.3 ± 2804.9	5154.4 ± 2181.6	5762.6 ± 2454.8	< 0.001
Hemoglobin (mg/dL)	13.3 ± 1.7	13.0 ± 1.7	11.9 ± 1.6	13.0 ± 1.7	0.058
Platelet (/mm^3)	147.0 ± 56.6	101.4 ± 54.0	82.2 ± 40.8	128.7 ± 59.8	< 0.001
Prothrombin time (INR)	1.1 ± 0.1	1.2 ± 0.2	1.6 ± 0.4	1.1 ± 0.2	< 0.001
AST (IU/L)	49.4 ± 58.6	79.1 ± 80.9	99.5 ± 111.2	62.0 ± 71.9	< 0.001
ALT (IU/L)	38.3 ± 42.4	46.5 ± 63.2	67.8 ± 149.5	42.4 ± 58.0	0.020
Total bilirubin (mg/dL)	0.8 ± 0.3	1.3 ± 0.8	4.6 ± 4.9	1.1 ± 1.3	< 0.001
Albumin (g/dL)	4.4 ± 0.3	3.5 ± 0.3	2.6 ± 0.3	4.0 ± 0.6	< 0.001
Serum AFP (IU/mL)	6.1 [3.0;39.2]	9.6 [4.8;35.2]	14.4 [6.1;106.3]	7.8 [3.5;38.3]	0.005
PIVKA-II (mAU/mL)	23.0 [17.0;34.0]	22.0 [16.0;35.0]	70.5 [28.0;143.5]	23.0 [17.0;36.5]	0.045
CTP grade					< 0.001
A	385 (99.7%)	189 (87.5%)	1 (4.0%)	575 (91.7%)	
B	1 (0.3%)	27 (12.5%)	24 (96.0%)	52 (8.3%)	
Neutrophil-lymphocyte ratio	4.2 ± 5.6	4.1 ± 8.1	3.7 ± 4.9	4.1 ± 6.5	0.933
BCLC stage					0.345
0	190 (49.2%)	95 (44.0%)	13 (52.0%)	298 (47.5%)	
A	191 (49.5%)	114 (52.8%)	12 (48.0%)	317 (50.6%)	
B	5 (1.3%)	7 (3.2%)	0 (0.0%)	12 (1.9%)	
mUICC stage					0.211
I	212 (54.9%)	112 (51.9%)	16 (64.0%)	340 (54.2%)	
II	162 (42.0%)	89 (41.2%)	8 (32.0%)	259 (41.3%)	
III	12 (3.1%)	15 (6.9%)	1 (4.0%)	28 (4.5%)	
Maximal tumor size (cm)	1.9 ± 0.6	2.0 ± 0.6	1.8 ± 0.6	1.9 ± 0.6	0.604
Sum of tumor size (cm)	2.1 ± 0.9	2.3 ± 0.9	2.0 ± 0.9	2.2 ± 0.9	0.139
Tumor number					0.073
1	331 (85.8%)	172 (79.6%)	24 (96.0%)	527 (84.1%)	
2	49 (12.7%)	39 (18.1%)	0 (0.0%)	88 (14.0%)	
3	6 (1.6%)	5 (2.3%)	1 (4.0%)	12 (1.9%)	
Encapsulated tumor	82 (21.2%)	43 (19.9%)	4 (16.0%)	129 (20.6%)	0.785
Subcapsular tumor	151 (39.1%)	77 (35.6%)	7 (28.0%)	235 (37.5%)	0425
Recurrence	183 (47.4%)	128 (59.3%)	16 (64.0%)	327 (52.2%)	0.010
Local tumor progression	44 (11.4%)	19 (8.8%)	2 (8.0%)	65 (10.4%)	0.558
Intrahepatic metastasis	177 (45.9%)	121 (56.0%)	13 (52.0%)	311 (49.6%)	0.055
Extrahepatic metastasis	5 (1.3%)	7 (3.2%)	3 (12.0%)	15 (2.4%)	0.002
Follow-up duration (days)	1546.8 ± 917.3	1314.6 ± 893.1	1034.0 ± 796.4	1437.9 ± 914.5	0.001

Data were expressed as number (percentage) or mean ± standard deviation. AFP and PIKV-II levels were presented as median [25%-75% interquar-tile range]. HBV, hepatitis B virus; HCV, hepatitis C virus; NASH, non-alcoholic steatohepatitis; WBC, white blood cells; INR, international normalized ratio; AST, aspartate aminotransferase; ALT, alanine aminotransferase; AFP, alpha-fetoprotein; PIVKA-II, protein induced by vitamin K absence or antagonist-II; CTP grade, Child-Turcotte-Pugh grade; BCLC, Barcelona Clinic Liver Cancer; mUICC, modified Union for International Cancer Control; ALBI, albumin-bilirubin.

Supplementary Table S2. Clinical information related to the RFA procedure.

Characteristics	No recurrence (n = 303)	Recurrence (n = 333)	Total (n = 636)	P value
Tumor location				0.779
S1	0 (0.0%)	1 (0.3%)	1 (0.2%)	
S2, 3	32 (10.6%)	38 (11.5%)	70 (11.1%)	
S4	39 (12.9%)	44 (13.3%)	83 (13.1%)	
S5, 6, 7, 8	231 (76.5%)	248 (74.9%)	479 (75.7%)	
Mean ablation time (min)	12.0 ± 5.9	13.7 ± 6.4	12.8 ± 6.2	0.001
RF needle puncture				0.578
1	280(92.4%)	302 (90.7%)	582 (91.5%)	
2	17 (5.6%)	25 (7.5%)	42 (6.6%)	
3	4 (1.3%)	5 (1.5%)	9 (1.4%)	
4	2 (0.7%)	1 (0.3%)	3 (0.5%)	
Post RFA complication	38 (12.5%)	40 (12.0%)	78 (12.3%)	0.934
Fever	18 (5.9%)	22 (6.6%)	40 (6.3%)	
Pleural effusion	19 (6.3%)	13 (3.9%)	32 (5.0%)	
Hemorrahge or hematoma	2 (0.7%)	4 (1.2%)	6 (0.9%)	
Abscess formation	2 (0.7%)	1(0.3%)	3 (0.5%)	
Cholecystitis	1 (0.3%)	2 (0.6%)	3 (0.5%)	
Others	0 (0.0%)	1 (0.3%)	1 (0.2%)	
Ablation zone area, sum (cm^2)	13.44 ± 9.39	12.38 ± 6.28	12.88 ± 7.93	0.097
Use of artifical ascites	131 (43.2%)	90 (27.1%)	221 (34.8%)	< 0.001
Repeat ablation for incomplete ablation	2 (0.7%)	8 (2.4%)	10 (1.6%)	0.148

Data were expressed as number (percentage) or mean ± standard deviation. RFA, radiofrequency ablation.
